# Supplementary material for: AI-HOPE-TP53: A Conversational Artificial Intelligence Agent for Pathway-Centric Analysis of TP53-Driven Molecular Alterations in Early-Onset Colorectal Cancer
Source: Cancers (Basel). 2025 Aug 31;17(17):2865. doi: 10.3390/cancers17172865 (PMC12427220; doi:10.3390/cancers17172865)
Supplement: Supplementary file 1 [file cancers-17-02865-s001.zip › cancers-3826781-supplementary.pdf]

cancers

MDPI

Supplementary Materials

Ei-Wen Yang, Brigette Waldrup and Enrique Velazquez-Villar-realAI-HOPE-TP53: A Conversational Artificial Intelligence Agent for Pathway-Centric Analysis of TP53-Driven Molecular Alterations in Early-Onset Colorectal Cancer

Ei-Wen Yang, Brigette Waldrup and Enrique Velazquez-Villarreal

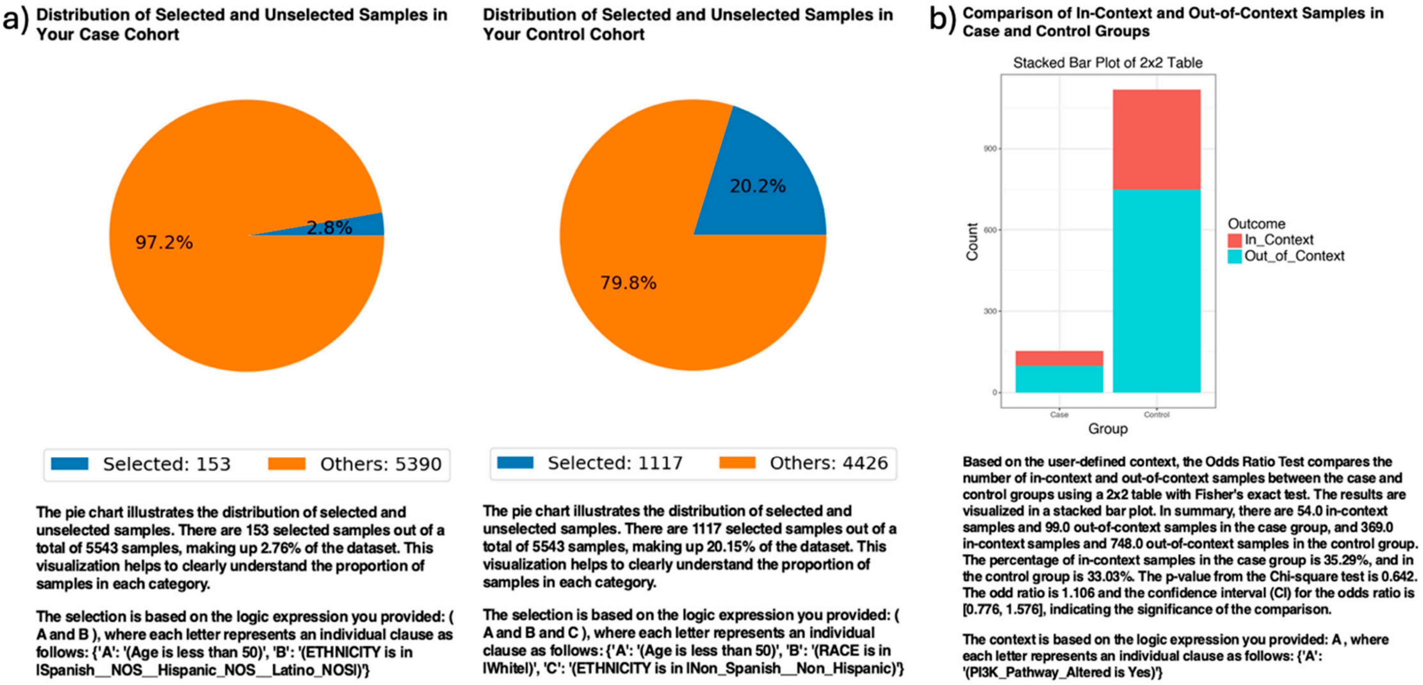

Figure S1. AI-HOPE-TP53 analysis of TP53 pathway alterations in early-onset colorectal cancer (EOCRC) among Hispanic/Latino (H/L) vs. Non-Hispanic White (NHW) patients. a) Pie charts illustrate the proportion of selected samples after applying natural language query filters. The case cohort (H/L) includes 153 EOCRC patients under age 50 with H/L ethnicity, representing 2.8% of the total dataset. The control cohort (NHW) includes 1,117 EOCRC patients under age 50 who are NHW, accounting for 20.2% of the queried population. b) A 2×2 odds ratio analysis compares the frequency of TP53 pathway alterations between the two cohorts. The stacked bar plot depicts the distribution of in-context (alteration present) and out-of-context (alteration absent) samples for both groups. TP53 pathway alterations were observed in 90.2% of H/L samples and 85.05% of NHW samples. The resulting odds ratio was 1.62 (95% CI: 0.926–2.825), with a p-value of 0.114, indicating a non-significant trend toward higher TP53 alteration frequency in H/L patients.

 https://doi.org/10.3390/cancers17172865

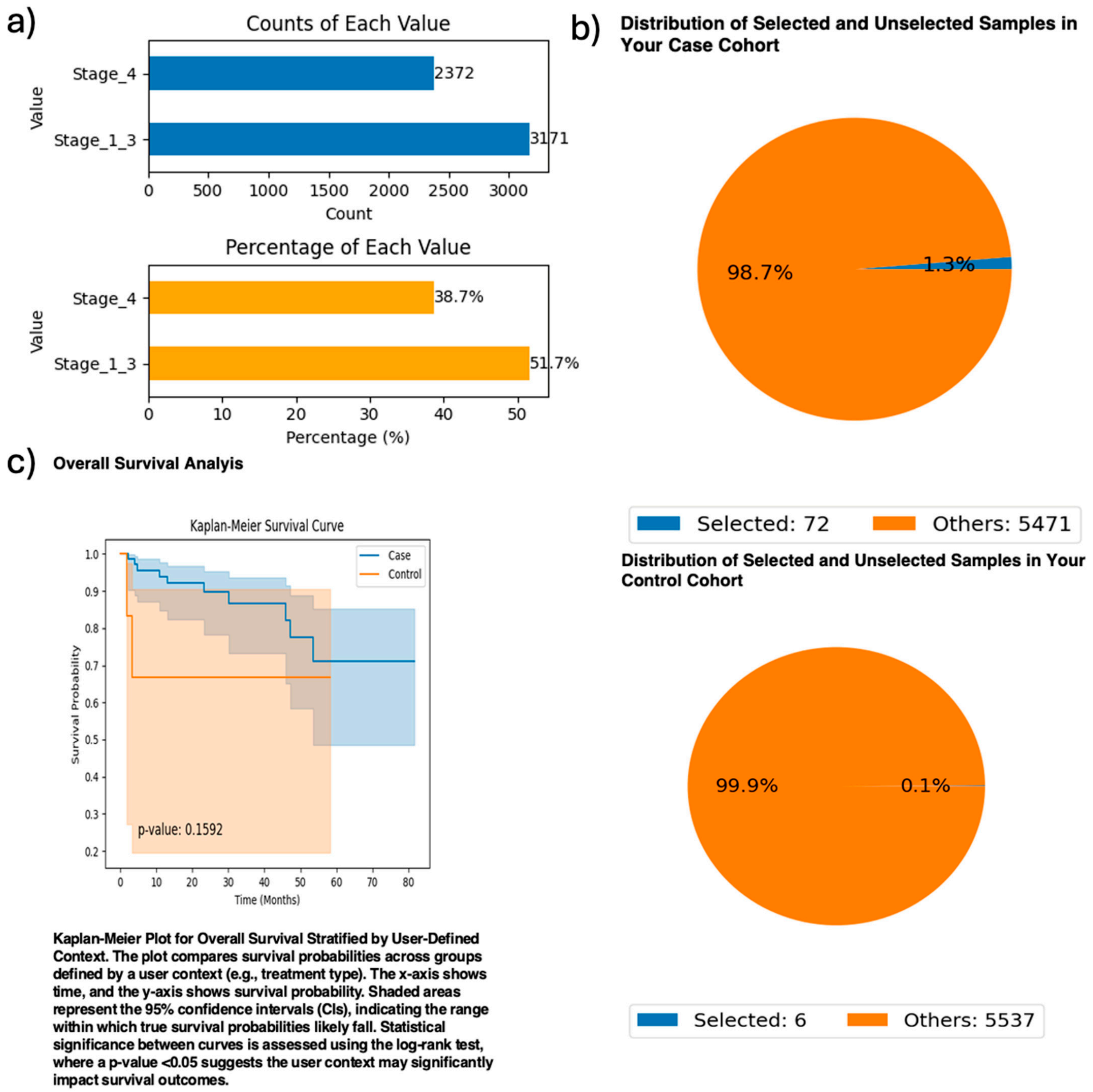

Figure S2. AI-HOPE-TP53 Analysis of CHEK1-Mutant Colorectal Cancer (CRC) Patients by Tumor Stage (Stage I–III vs. Stage IV). This figure illustrates the application of AI-HOPE-TP53 to evaluate survival outcomes among CRC patients harboring CHEK1 mutations, stratified by disease stage. The case cohort includes patients with early-stage tumors (Stage I–III), while the control cohort comprises patients with advanced-stage tumors (Stage IV). a) Bar plots display the dataset-wide distribution of clinical stage categories. Stage I–III cases (labeled as “Stage_1_3”) represent 51.7% of the total dataset (n = 3,171), while Stage IV cases account for 38.7% (n = 2,372). The top panel shows absolute sample counts, and the bottom panel shows proportional representation. b) Pie charts illustrate the cohort selection process based on natural language query filters. The early-stage CHEK1-mutated cohort includes 72 selected samples (1.3% of the dataset), while the advanced-stage CHEK1-mutated control group includes 6 samples (0.1%). These visuals highlight the rarity of CHEK1 mutations in stage-stratified CRC subgroups. c) Kaplan-Meier survival analysis compares overall survival between early-stage and late-stage CHEK1-mutated CRC patients. Although early-

stage patients show a trend toward improved survival, the difference was not statistically significant (p = 0.1592). Shaded regions represent the 95% confidence intervals.

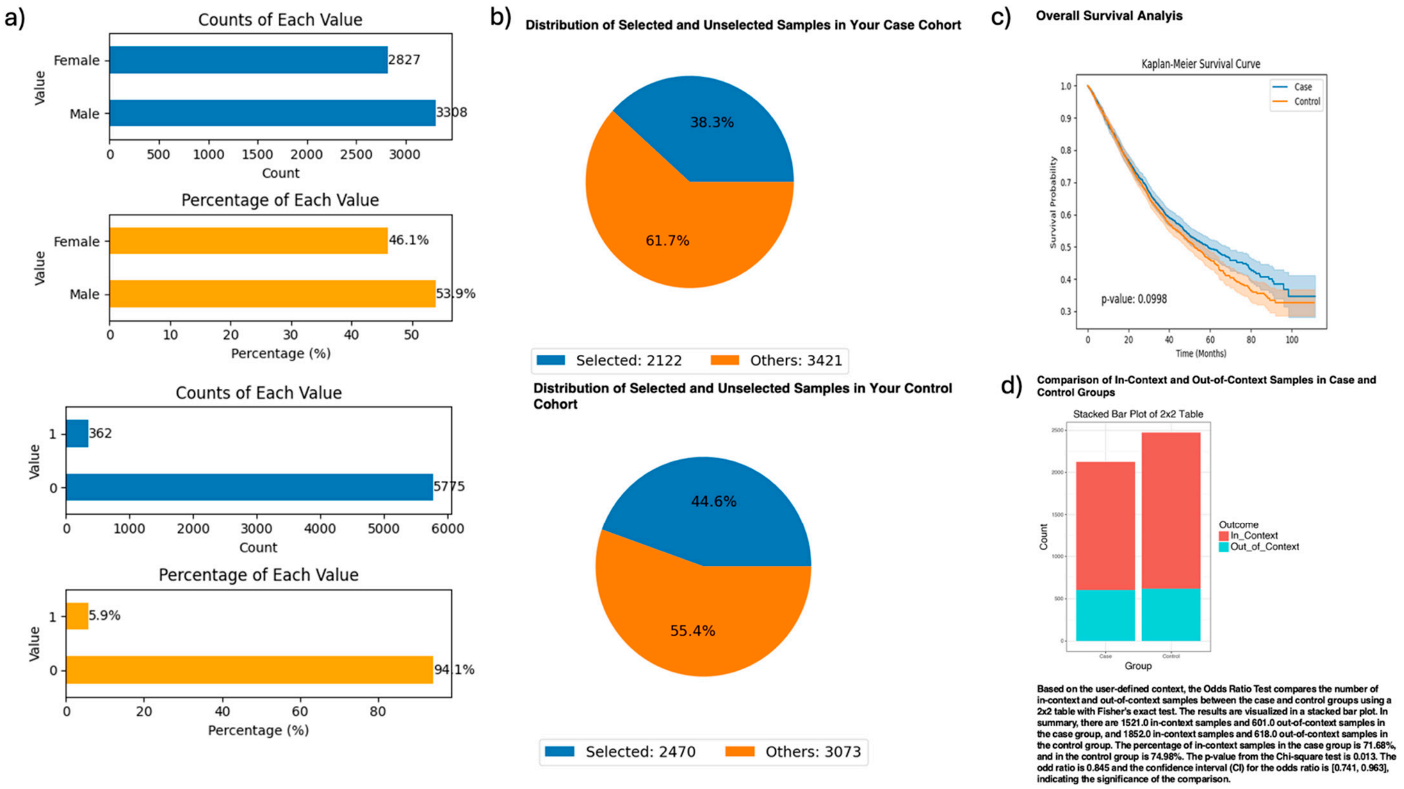

Figure S3. AI-HOPE-TP53 Analysis of Gender-Based Survival in TP53 Pathway–Altered Colorectal Cancer (CRC) Patients Receiving FOLFOX Chemotherapy. This figure demonstrates the use of AI-HOPE-TP53 to analyze sex-based differences in clinical outcomes among CRC patients with TP53 pathway alterations, contextualized by receipt of standard FOLFOX chemotherapy (Fluorouracil, Leucovorin, Oxaliplatin). a) Bar plots summarize baseline cohort characteristics. The top panel illustrates gender distribution across the dataset, with males (n = 3,398; 53.9%) slightly outnumbering females (n = 2,827; 46.1%). The bottom charts display counts and proportions of TP53 pathway–altered samples receiving FOLFOX treatment (Yes = 362, No = 5,775), highlighting the relative rarity of this mutation-treatment context. b) Pie charts depict the number of selected samples after applying the user-defined natural language query. The case cohort (females with TP53 pathway alterations) includes 2,122 samples (38.3%), while the control cohort (males with TP53 pathway alterations) includes 2,470 samples (44.6%). These visuals reflect near-equivalent representation and support gender-stratified comparative analysis. c) Kaplan-Meier survival analysis compares overall survival between female and male patients with TP53 pathway–altered CRC undergoing FOLFOX chemotherapy. Although the curves suggest a trend toward improved survival in female patients, the difference did not reach statistical significance (p = 0.0998). Confidence intervals overlap throughout the follow-up period. d) A 2×2 odds ratio analysis contextualized by FOLFOX exposure evaluates the enrichment of in-context samples (defined by gender and TP53 pathway mutation) between cohorts. The stacked bar plot shows that 71.68% of female samples and 74.96% of male samples met the full query context. The odds ratio was 0.845 (95% CI: [0.741, 0.963]; p = 0.0138), suggesting a statistically significant underrepresentation of FOLFOX-treated TP53 pathway–altered female patients relative to their male counterparts.

Table S1. Summary of AI-HOPE-TP53 Analyses in Colorectal Cancer.

Analysis	Comparison	Outcome	Statistical Result	Significance
Ethnicity-stratified TP53 alterations (EOCRC, colon only)	H/L vs. NHW	Higher prevalence in H/L (91.46% vs. 83.39%)	OR = 2.13; 95% CI: 0.956–4.767; p = 0.084	Not significant (trend)
Ethnicity-stratified TP53 alterations (EOCRC, all sites)	H/L vs. NHW	Higher prevalence in H/L (90.2% vs. 85.05%)	OR = 1.62; 95% CI: 0.926–2.825; p = 0.114	Not significant (trend)
Ethnicity-specific survival (TP53-mutant CRC)	H/L vs. NHW	Trend toward improved survival in H/L	p = 0.1141	Not significant (trend)
Tumor subsite survival (ATM-mutant CRC)	Colon vs. Rectum	Trend toward poorer survival in colon tumors	p = 0.3134	Not significant (trend)
Age-stratified survival (TP53-mutant, FOLFOX-treated CRC)	Early-onset (<50) vs. Late-onset (≥50)	Improved survival in early-onset group	p = 0.0149	Significant
Ethnic enrichment (TP53-mutant, FOLFOX-treated CRC)	H/L representation, early- vs. late-onset	H/L overrepresentation in early-onset	OR = 2.002; 95% CI: 1.524–2.632; p < 0.0001	Significant
Stage-specific survival (CHEK1-mutant CRC)	Stage I–III vs. Stage IV	Trend toward longer survival in early-stage	p = 0.1592	Not significant (trend; small N)
Gender-based survival (TP53-altered, FOLFOX-treated CRC)	Female vs. Male	Trend toward improved survival in females	p = 0.0998	Not significant (trend)
Gender-based treatment representation (TP53-altered, FOLFOX-treated CRC)	Female vs. Male	Females underrepresented in FOLFOX treatment	OR = 0.845; 95% CI: 0.741–0.963; p = 0.0138	Significant

Abbreviations: OR, odds ratio; CI, confidence interval; p, p-value; N, sample size; EOCRC, early-onset colorectal cancer; H/L, Hispanic/Latino; NHW, Non-Hispanic White; CRC, colorectal cancer.
